# Impaired Perinatal Growth and Longevity: A Life History Perspective

**DOI:** 10.1155/2009/608740

**Published:** 2009-09-06

**Authors:** Deborah M. Sloboda, Alan S. Beedle, Cinda L. Cupido, Peter D. Gluckman, Mark H. Vickers

**Affiliations:** Liggins Institute, National Research Centre for Growth and Development, The University of Auckland, Private Bag 92019, Auckland 1142, New Zealand

## Abstract

Life history theory proposes that early-life cues induce highly integrated responses in traits associated with energy partitioning, maturation, reproduction, and aging such that the individual phenotype is adaptively more appropriate to the anticipated environment. Thus, maternal and/or neonatally derived nutritional or endocrine cues suggesting a threatening environment may favour early growth and reproduction over investment in tissue reserve and repair capacity. These may directly affect longevity, as well as prioritise insulin resistance and capacity for fat storage, thereby increasing susceptibility to metabolic dysfunction and obesity. These shifts in developmental trajectory are associated with long-term expression changes in specific genes, some of which may be underpinned by epigenetic processes. This normative process of developmental plasticity may prove to be maladaptive in human environments in transition towards low extrinsic mortality and energy-dense nutrition, leading to the development of an inappropriate phenotype with decreased potential for longevity and/or increased susceptibility to metabolic disease.

## 1. Introduction

Life history biology describes patterns of growth, reproduction, and aging, and for any particular species evolution has acted to optimize allocation of limited resources among these components of life history so as to maximize fitness (reproductive success) [1, 2]. Since Darwinian selection acts to optimize fitness, and under most conditions, an organism's lifespan is determined by extrinsic mortality (e.g., predation), there is, in general, little selective pressure for postreproductive longevity, except perhaps for the *grandmother effect*; the hypothesized benefit to kin conferred by a long postmenopausal lifespan in human females [3]. It is only under conditions of reduced extrinsic mortality, as in the protected environments typical of laboratory organisms or of most modern human populations, that intrinsic mortality (aging) becomes biologically relevant. 

 Aging may be actualized by (i) accumulation of somatic mutations or toxic injury to DNA and cellular proteins and organelles with deleterious effects in later life; (ii) antagonistic pleiotropy favoring alleles with beneficial effects early in life but with negative influences on health in older age; (iii) life history strategies resulting in resource allocation to early growth and reproduction rather than to tissue repair processes that enhance longevity [4]. At the level of an individual organism, developmental plasticity, the ability to develop differently depending on the particular environment, is a well-established concept in comparative biology [5]. A large literature describes how early life events, often but not inevitably resulting in impaired fetal growth, can have profound effects on later health in humans, increasing susceptibility to chronic noncommunicable diseases such as obesity and type 2 diabetes [6] and increasing the risk of premature adult death [7]. Developmental plasticity has been proposed to be the mechanism underpinning these effects [8]. 

 In this brief review, we describe how understanding the interface between life history biology and individual development can contribute to our knowledge of chronic disease and aging in humans.

## 2. Nutrition and Aging

A common theme in aging-related research is the lifespan-increasing effect of calorie restriction in organisms ranging from yeast to mammals [9]. Nutritional cues might have quite distinct effects depending on when they act, and in mammals it appears important to distinguish between nutritional cues acting in periconceptional, fetal, or early neonatal life, and those acting later after weaning. In laboratory rodents, postmaturational calorie restriction increases longevity [9]. In sharp contrast, *prenatal* calorie restriction induces a range of adverse consequences in later life, including metabolic dysfunction, cardiovascular and renal pathophysiology, impaired neurodevelopment [10], and shortened lifespan [11–13]. Protein restriction during early life has been shown to influence key factors regulating oxidative stress pathways as well as insulin signalling [14, 15]. Accelerated growth after an earlier period of nutritional limitation, also referred to as catchup growth, appears particularly deleterious [13], and its prevention by postnatal nutritional restriction prevents the adverse effects of earlier undernutrition [12]. These data, however, are not always supported by human evidence. Although there is a relative paucity of data in human cohorts, effects of prenatal nutritent restriction appear to be dependent upon the duration and the severity of the famine such as those highlighted by the Dutch Famine (1944-1945) and the Finnish Famine during the 1860s [16, 17].

## 3. Aging in Key Systems

### 3.1. Reproduction

Age at menarche and/or puberty are markers of reproductive maturation. Literature shows an association between adversity early in development and accelerated maturation of the reproductive axis; low birth weight girls show early age at menarche and advanced puberty [18–20]. Longitudinal studies have demonstrated that small for gestational age (SGA) girls have reduced uterine size and ovarian volume, with persistent elevation of follicle-stimulating hormone, increased levels of luteinising hormone and fasting insulin, and an excess of abdominal fat [19]. Experimentally, we have observed that offspring of rats poorly nourished throughout gestation have advanced puberty [21].

### 3.2. Metabolism

Aging in humans is associated with progressive impairment of insulin secretion [22–24], compounded by the loss of lean muscle mass and an increase in central adiposity that occurs with age [25], both of which contribute to impaired insulin action. In addition to aging, alterations in insulin sensitivity are further modified by complex interactions between the degree of obesity, gender, and physical activity. Both reduced insulin secretion and sarcopenia are amplified in elderly humans by poor early growth [26, 27]. Thus, a substantial proportion of the comorbidity and mortality that occur with type 2 diabetes and the metabolic syndrome, in particular cardiovascular disease, may directly or indirectly relate to the changes in insulin secretion and action that evolve with aging and that are exacerbated by early life adversity. 

 These findings are supported by numerous studies in laboratory animal models. For example, prenatally undernourished rats develop central obesity and reduced skeletal muscle mass in adulthood, together with altered insulin sensitivity and altered appetite regulation [28, 29].

### 3.3. Musculoskeletal System

Among the elderly, pathological loss of density in bone, that is, osteoporosis, has become a major public health problem in developed countries. Osteoporosis is a multifactorial skeletal disorder characterized by low bone mass and microarchitectural deterioration of bony tissue, with a consequent increase in the risk of fracture. The bone mass of an individual in later life depends upon the peak obtained during skeletal growth and the subsequent rate of bone loss. Available genetic markers are only able to explain a small proportion of the variance in individual bone mass or fracture risk [30], and evidence is now accumulating that osteoporosis risk may be influenced by environmental influences during critical periods of early development. Studies have shown that growth in infancy correlates with adult bone mass and strength independently of adult lifestyle [31, 32] and that growth in infancy and childhood is a predictor of the risk of later hip fracture [33]. More recent reports highlight the central role of maternal nutrition as a predictor of postnatal bone development and bone mineral density [34, 35], although followup of aged adults still remains to be done.

### 3.4. Oxidant Systems

Several studies of the impact of early life environmental factors have suggested that changes in oxidant defences may be involved in accelerated age-related pathology. Hypertension in adult rat offspring after maternal low protein diet is associated with increased oxidative stress, and both are prevented by maternal administration of a lipid peroxidation inhibitor [36]. The effects of fetal growth retardation on the kidney are exacerbated by rapid postnatal growth, which is associated with shortened renal telomeres and reduced life span [37, 38] and with mitochondrial abnormalities and renal coenzyme Q deficiency [39]. Conversely, protein restriction during lactation, which decreases early neonatal growth, is nephroprotective, increases renal telomere length, improves antioxidant status and insulin sensitivity, and increases longevity [13, 40]. In humans, there is increased oxidative stress in prepubertal SGA children [41]. Other observations associate oxidative stress with beta cell failure [42] and with impaired glucose signalling in the pancreas [43, 44].

## 4. Early Life Events and Implications for Aging

There is a growing recognition that environmental influences acting during windows in development induce long-lasting structural and functional changes underpinned at least in part by molecular epigenetic processes [45, 46]. Such changes, not restricted to parentally imprinted genes, may underlie the well-established effects of the early life environment on the incidence of noncommunicable diseases in humans [6], effects mirrored and well studied in animal models [10, 47]. Emerging concepts in evolutionary-developmental biology and associated life history theory [1, 2, 5] provide underlying principles to aid interpretation of these findings. (i) Organisms, including humans, evolved to maximize (reproductive) fitness, not longevity: all organisms with separate somatic and germ cell lines have evolved by natural selection to die and thus face tradeoffs particularly between fertility, mortality, and the mortality of their offspring. Particular solutions to these tradeoffs evolved for different species and within species facing variable environments. For example, tradeoffs between lifespan and fecundity have been demonstrated in model organisms [48] as well as in humans [49, 50]. (ii) Life history consists of age-specific schedules of mortality and fecundity as well as traits that are directly the result of such schedules (e.g., puberty) or are directly connected to them (e.g., growth, body size). Life history traits correlate with each other in ways that constrain the possible sets of relationships. These constraints are determined by tradeoff decisions in response to the energetic environment. Such decisions may have irreversible consequences because of temporal and associative constraints in development. Developmental plasticity is limited to that period when the costs of maintaining plasticity to respond to variable environments outweigh the benefits. (iii) Natural selection should result in an optimal allocation pattern, given these constraints, but in addition to genetic determinants of life history tradeoffs, there are developmental and epigenetic modifiers. 

 Thus, mechanisms by which perinatal nutrition affects later aging phenomena can be interpreted as the result of developmentally induced tradeoffs leading to acceleration of the tempo of maturation under perceived conditions of an actual or predicted threatening environment, so that the probability of early reproductive success is increased despite tradeoffs that manifest as late life costs. The organism reprioritizes investment into early growth and reproductive maturation at the expense of organs that are less critical for such purposes (brain, kidney) and of general tissue repair [51]. Thus, for example, acceleration of reproductive maturation by early life adverse conditions has been shown in laboratory animals (described earlier), in cohort studies on human females [18, 20] and by anthropological observations [50], although birthweight data were not central to these observations. Crucially, such accelerated maturation is contingent on adequate nutrition in childhood [52]. In extreme circumstances, advances in menarche are seen in girls from deprived backgrounds adopted into developed countries [53].

## 5. Leptin as a Life History Regulator

There is growing evidence that leptin has a broad and integrative role in mammalian physiology, acting as a signal of energy balance to synchronize growth and fertility with periods of food availability [54]. Leptin, a 16 kDa protein predominantly derived from adipocytes, signals energy sufficiency by means of central and peripheral mechanisms that reduce appetite and increase energy expenditure. Leptin acts centrally, via specific leptin receptors on neurons in the arcuate nucleus of the hypothalamus, to modulate appetite, thermogenesis, and metabolism via second-order neurons [55]. Studies have demonstrated that leptin acts as a neurotrophic factor that promotes maturation of these circuits via promotion of projections from the arcuate nucleus [56, 57]. It appears that in neonatal rodents, these connections are established soon after birth. Neonatal rats show a leptin surge that is maximal at 10–12 days of life, and it has been shown that this surge may drive appetite regulating neuronal connections that are fully functional by 21 days of life (weaning) [58]. Indeed, in offspring subject to prenatal undernutrition that later develop metabolic dysfunction and obesity, the leptin surge is altered and attenuated [59, 60], providing further evidence for leptin in the neonatal period as a determinant of later energy balance. 

 Leptin also has effects on the hypothalamo-pituitary-gonadal axis in developing rodents, regulating secretion of gonadotropins, and appears to be a permissive factor in the onset of puberty as well as in menstrual/estrus cycle regulation [61, 62]. As would be expected from its permissive effect on the gonadotropic axis, postweaning treatment with exogenous leptin accelerates puberty in rodents [63]. Leptin appears to act indirectly through its regulation of other factors such as the recently identified KiSS-1 gene product kisspeptin [62]. 

 Leptin administration during the neonatal period affects energy metabolism in later life. In our rat model of metabolic programming [29, 64], neonatal leptin administration completely prevented the later development of metabolic dysfunction [65, 66] and reversed the associated long-term changes in gene expression and epigenetics [67, 68]. However, the mechanisms underlying these observations require further investigation at the level of the hypothalamus as previously suggested [69] in addition to examination of regional fat deposition. Intriguingly, these leptin effects at the level of gene expression and promoter methylation in the liver were directionally dependent upon the nutritional status of the mother, leading us to the hypothesis that maternal undernutrition induces a developmental bifurcation in the metabolic trajectory, suggesting effects on life history traits [68]. Endogenous leptin in the neonate, reflecting infant fat mass and perhaps maternal nutritional state, therefore, may act as an endogenous regulator of the balance of life history traits including aging. Exogenous leptin, if active within this window of metabolic sensitivity, can override prior cues suggesting against a threatening environment in favour of a safer environment and thus bias the organism towards longevity. Leptin, because it reflects fat mass, is a signal with a degree of inertia and thus is likely to be read as an indicator of the nutritional environment over time rather than instantaneously and, therefore, is more suitable as a predictive signal [70]. Although it is difficult to translate from rodents to humans, animal models do provide important insights into possible therapeutic avenues to alter and improve developmental outcomes following an adverse early start to life.

## 6. Conclusion and Perspectives

We have suggested that programming (as used in the developmental origins of health and disease literature) is generally not a pathophysiological process, but rather a reflection of developmental cues acting to influence the processes of developmental plasticity [71]. While these processes may have an adaptive origin, namely, to match the developing organism to either its immediate or anticipated environment, they may have maladaptive consequences [45]. Developmental plasticity refers to the set of mechanisms by which environmental cues act in critical windows to induce a range of phenotypes for a given genotype, and we have suggested that such responses may be anticipatory, shifting the developmental trajectory so that the phenotype develops with a “design” to match the anticipated later environment [71]. We have termed this process a predictive adaptive response [72], a phenomenon extensively recognized in the comparative evolutionary-developmental biology literature [73] and in human biology [74]. The so-called developmental origins pathway, whereby being born smaller (albeit within the normal range of birth sizes) or whereby nutritional or hormonal cues act on the fetus or neonate (without necessarily impacting on growth), can be explained by these processes coupled with a mismatch between the predicted and actual mature environment [45]. 

 We suggest that a life history approach provides a coherent understanding of how these different processes interplay [75]. Developmental plasticity [8], underpinned at least in part by epigenetic processes affecting nonimprinted genes, allows the developing organism to “further tune” its development to its anticipated environment. As suggested by our studies with leptin [65, 68] and by other studies manipulating prenatal and postnatal nutrition [12, 13], developmental cues induce integrated shifts in a suite of traits. As the key cues in early development are likely to be nutritional or stress related, it is to be anticipated that this integrated shift in the balance of traits affects key elements of life history and that these interplay through a series of tradeoffs. Thus, to take the extremes, if the fetus or neonate predicts a nutritionally good environment, it can afford a balance of life history traits associated with investing for longevity and thus greater tissue reserve, repair, and antioxidant activity and for larger body mass and thus slower maturation. This is a strategy designed for optimal fitness in a nonthreatening environment. If the developing organism predicts a threatening environment, it must accelerate maturation, and thus obesity may appear in the prepubertal period [52]. Under these circumstances, the organism also invests less in tissue reserve (i.e., fewer neurons and nephrons) and antioxidant capability, less into body growth and bone density, reduces muscle mass, and induces a series of central and peripheral metabolic changes that allow it to store energy, when it is available, as fat. The organism will also reduce energy utilization through insulin resistance. This suite of adaptations will directly affect longevity by limiting tissue reserve and repair capacity in later life. Furthermore, if the future environment rather than being deprived actually features the ready availability of energy-dense foods on a background of low energy expenditure, as is typical of human societies that have undergone the nutrition transition [76, 77], then insulin resistance and enhanced fat storage capacity will promote the development of obesity and type 2 diabetes, further impacting on health and longevity.
